# Do disempowered childbearing women give birth at home in Sierra Leone? A secondary analysis of the 2019 Sierra Leone demographic health survey

**DOI:** 10.1186/s12884-023-06126-y

**Published:** 2023-11-22

**Authors:** Peter Bai James, George A Yendewa, Abdulai Jawo Bah, Augustus Osborne, Satta Sylvia Kpagoi, Emmanuel Kamanda Margao, Jia Kangbai, Jon Wardle

**Affiliations:** 1 National Centre for Naturopathic Medicine, Faculty of Health, Southern Cross University, Lismore, Australia; 2 Faculty of Pharmaceutical Sciences, College of Medicine and Allied Health Sciences, University of Sierra Leone, Freetown, Sierra Leone; 3 Department of Medicine, Case Western Reserve University School of Medicine, Cleveland, OH 44106 USA; 4 Division of Infectious Diseases and HIV Medicine, University Hospitals Cleveland Medical Center, Cleveland, OH 44106 USA; 5 Johns Hopkins Bloomberg School of Public Health, Baltimore, MD 21205 USA; 6 Institute for Global Health and Development, Queen Margaret University Edinburg, Musselburgh, Scotland UK; 7 Department of Biological Sciences, School of Environmental Sciences, Njala University, Njala Campus, Njala, Sierra Leone; 8 Bo Government Hospital, Ministry of Health and Sanitation, Bo, Sierra Leone; 9 School of Community Health Sciences, Njala University, Bo Campus, Bo, Sierra Leone; 10 Faculty of Health Sciences and Disaster Management, Eastern Technical University of Sierra Leone, Kenema, Sierra Leone

**Keywords:** Homebirth, Women’s empowerment, Sierra Leone

## Abstract

**Background:**

A nationwide assessment of the link between women’s empowerment and homebirth has not been fully examined in Sierra Leone. Our study examined the association between women’s empowerment and homebirth among childbearing women in Sierra Leone using the 2019 Sierra Leone Demographic Health Survey (2019 SLDHS) data.

**Method:**

We used the individual file (IR) of the 2019 SLDHS dataset for our analysis. A total of 7377 women aged 15–49 years who gave birth in the five years preceding the survey were included. Outcome variable was “home birth of their last child among women in the five years preceding the 2019 SLDHS. Women’s empowerment parameters include women’s knowledge level, economic participation, decision-making ability and power to refuse the idea of intimate partner violence. We used the complex sample command on SPSS version 28 to conduct descriptive and multivariate logistic regression analyses.

**Results:**

Three in every 20 women had home childbirth (n = 1177; 15.3%). Women with low [aOR 2.04; 95% CI 1.43–2.92] and medium [aOR 1.44; 95%CI 1.05–1.97] levels of knowledge had higher odds of giving birth at home compared to those with high levels of knowledge. Women who did not have power to refuse the idea of intimate partner violence against women were more likely to had given birth at home [aOR 1.38; 95% CI1.09-1.74]. In addition, women with no [aOR 2.71; 95% CI1.34-5.46) and less than four antenatal care visits [aOR 2.08; 95% CI:1.51–2.88] and for whom distance to a health facility was a major problem [aOR 1.95; 95% CI1.49-2.56] were more likely to have had a homebirth. However, no statistically significant association was observed between a women’s decision-making power and home birth [aOR 1.11; 95% CI 0.86–1.41].

**Conclusion:**

Despite improvements in maternal health indicators, homebirth by unskilled birth attendants is still a public health concern in Sierra Leone. Women with low knowledge levels, who did not have power to refuse the idea of intimate partner violence against women, had less than four ANC visits and considered distance to a health facility as a major problem had higher odds of giving birth at home. Our findings reflect the need to empower women by improving their knowledge level through girl child and adult education, increasing media exposure, changing societal norms and unequal power relations that promote gender-based violence against women, and improving roads and transport infrastructure.

**Supplementary Information:**

The online version contains supplementary material available at 10.1186/s12884-023-06126-y.

## Background

Maternal mortality is still a public health concern in low-income countries, especially sub-Saharan Africa (sSA), despite the interest, commitments and resources that have been put forward to reducing its incidence by global health partners, governments and local health authorities. Globally, 94% of all maternal deaths occur in low-income countries, with sSA and Southern Asia accounting for 86% [1]. SSA bears the greater burden of maternal deaths among these two worst-performing regions. Current estimates suggest that 542 maternal deaths per 100 000 live births occurred in sSA, and the lifetime risk of maternal death is 1 in 37 compared to 1 in 7800 in Australia and New Zealand [1]. Homebirth without a skilled birth attendant has been identified as a significant factor contributing to the high maternal mortality ratios (MMRs) in sSA. Childbirth in the presence of a skilled birth attendant who can identify and manage uncomplicated labour and birth, identify and treat complications, and provide primary care and referral, has been promoted as a strategy to reduce the high maternal mortality ratio in sSA [2]. Presence of a skilled birth attendant during labour and childbirth has been associated with decreased maternal mortality and increased newborn survival [3, 4]. The number of births, however, occurring by skilled health personnel in sSA is relatively low despite progress made over the years [5, 6].

Sierra Leone is known to be one of the countries in sSA with the highest MMR, with the current estimate putting it at 1120 maternal deaths per 100 000 live births. Thirty-six per cent of all deaths amongst women aged 15–49 years are attributed to maternal death, and postpartum haemorrhage (46%), hypertension (22%), obstructed labour (21%) and sepsis (11%) are considered the leading causes of maternal deaths [7]. Since implementing the free healthcare policy in 2010 for pregnant women, lactating mothers and underfive children, Sierra Leone has seen an improvement in most of its maternal and child health indicators, such as antenatal care coverage and skilled birth attendants [8, 9]. Yet still, the lifetime risk of maternal death in Sierra Leone remains among the highest in the world [1]. Such mismatch in care utilisation and outcome has been attributed to the quality of care provided to pregnant women, lactating mothers and under five children. Recent reports highlight high misdiagnosis and nonadherence to standard treatment guidelines and suggest that most maternal deaths occur within healthcare facilities [10].

In line with the World Health Organisation’ (WHO) recommendation, the current national Reproductive, Maternal, Newborn, Child and Adolescent Health Policy and strategic plan of Sierra Leone promotes facility-based childbirth [7]. Although progress has been made over the years, a substantial number of women still prefer homebirth, many of which are attended by unskilled birth attendants [11, 12]. Long distances from health facilities, cultural norms, poor quality and disrespectful care have been identified as reasons for women’s preference for homebirth over institutional childbirth [13–15]. Studies from other sSA countries have identified sociodemographic, obstetric and empowerment predictors of homebirth [5, 16–21]. In the context of health, women’s empowerment can be considered as women’s ability to make informed decisions about their health and wellbeing. Empowerment may include their socioeconomic status, household-decision making power and stance regarding gendered-based violence [22, 23]. A qualitative study in rural Sierra Leone found that even though women were custodians of family finances and involved in income-generating activities as a form of financial independence/empowerment, health-related decision-making power, especially when it relates to pregnancy and childbirth, was still in the hands of men [24]. Studies conducted in other sSA countries have identified women’s empowerment in the form of knowledge level, economic participation, decision-making power and attitude towards gender-based violence as predictors of maternity care utilisation [5, 25, 26].

Studies of women’s choice of place of birth in Sierra Leone have either employed a qualitative study design or are subnational and not representative of the whole Sierra Leonean population [14, 15, 24, 27–29]. Recent national studies have examined determinants of skilled birth attendants use [30]. Although a recently published study has explored the link between skilled birth attendants use and women’s empowerment, it only focussed on married women [31]. Secondly, the study did not capture a key women’s empowerment parameter, i.e., attitude towards intimate partner violence (IPV) against women [31]. To our knowledge, no nationwide study has examined the association between women’s empowerment and homebirth among childbearing women, regardless of their marital status in Sierra Leone. Secondly, the association between women’s choice of homebirth and their attitude towards the idea of IPV against women(power to refuse or not refuse the idea of IPV against women), a key women’s empowerment parameter, has not been examined either. Thus, our study examined the association between women’s empowerment and homebirth among childbearing women in Sierra Leone using the 2019 Sierra Leone Demographic Health Survey.

## Methods

### Data sources and sampling

We used the Sierra Leone 2019 Demographic Health Survey (2019 SLDHS) as our secondary data source for analysis. The 2019 SLDHS is a national cross-sectional survey with a sample of 13,872 selected households designed to provide reliable estimates for key population and health indicators at the national and subnational level [12]. A stratified, two-stage cluster sampling design was employed and the sampling frame for the survey was derived from the 2015 Sierra Leone Population and Housing Census [12]. The first stage involved selection of 578 enumeration areas (EAs) based on probability proportional to size (214 EAs in urban and 364 EAs in rural areas). In the second stage, 24 households were selected from each EAs using a systematic sampling method. A total of 5,564 urban and 8,308 rural households were selected, while 16.099 women (15–49 years of age) and 7.429 men (15–59 years of age) were eligible to be interviewed [12]. The 2019 SLDHS used standardised and country-specific questionnaire modules that collected information on specific population and health issues. A complete description of the sampling procedure can be accessed in the recent 2019 SLDHS report [12]. We used the women’s recoded file (IR) for analysis. Out of the 16.099 eligible women, 15.574 completed the interviews at a response rate of 96.7%. Data were used from 7.377 women aged 15–49 years who gave birth in the five years preceding the survey.

### Outcome variable

Our outcome variable was “home birth of their last child among women aged 15–49 years in the five years preceding the 2019 SLDHS”. Women were asked in the 2019 SLDHS where they gave birth during their last childbirth. The responses were “her home”, “other home”, “government hospital”, “government health centre”, “government health post”, “other public sector”, private hospital/clinic”, “other private medical sector” and “others”. Based on previous studies, we grouped all responses into two groups [16, 19, 26]. The “homebirth group” constituted “home” and “other home” responses as they involved births that occurred outside the health facility setting. The “health facility group” constituted “government hospital”, “government health centre”, “government health post”, “other public sector”, private hospital/clinic”, “other private medical sector” and “others” since they signify birth in health facilities. We recoded the “home birth group” as “Yes = 1” and the “health facility group” as “No = 0”.

### Independent variables

The main independent variable was women’s empowerment. In line with previous studies [5, 25, 32] women’s empowerment included: women’s knowledge level (comprising listening to radio, reading newspaper/magazine, watching television, internet access and maternal educational status), economic participation-currently working (Yes vs. No), decision-making ability (constituting decision making about her health, purchasing large household items and visiting family/relatives), attitude towards the idea of IPV against women (“having the power to refuse or not: beating if wife goes out without telling husband”, “beating if wife neglects the children”, “beating if wife argues with husband”, “beating if wife refuses to have sex with husband”, beating if wife burns the food) [5, 25, 32]. Regarding decision-making ability, we coded “yes” if the woman was involved in any of the decision-making parameters, alone or with her husband or someone else; “no” if she was not involved in any of the three decision-making parameters. Regarding the power to refuse the idea of IPV against women, we assessed and considered “having the power to refuse” if the respondent was against all of the following five statements “beating justified if wife goes out without telling husband, “beating justified if wife neglects the children”, “beating justified if wife argues with husband”, “beating justified if wife refuses to have sex with husband”, beating justified if wife burns the food and “not having the power to refuse” if she agrees or not sure of any of the aforementioned five wife beating statements. We recoded women’s knowledge level, decision-making ability and women’s power to refuse the idea of IPV into low, medium and high based on previous studies [5, 25].

We included the following sociodemographic and obstetric explanatory variables based on previous studies and their theoretical significance in influencing homebirth [5, 16–19, 21, 26, 33–35]. The independent variables included maternal age (15–19, 20–24, 25–29, 30–34, 35–39, 40–44, 45–49), region (Eastern, Northern, North-Western, Southern and Western), residence (urban and rural), religion (Christianity, Islam and None), husband/partner’s education level (no formal education, primary school, secondary school, higher), sex of household head (male vs. female), parity (1–5 vs. > 5), ever had a terminated pregnancy (Yes vs. No), wanted last child (wanted then, wanted later, wanted no more), antenatal visit (no antenatal visit, 1–3 visits, 4–7 visits and eight or more visits), getting medical help for the woman herself: getting permission to go to the hospital (not a problem, not a big problem and big problem); getting the money needed for treatment (not a problem, not a big problem and big problem); distance to health facility (not a problem, not a big problem and big problem) and covered by health insurance (Yes vs. No). Husband/partner’s occupation was coded as “Not working/Don’t know” and working (sales, clerical and services, agricultural-self-employed, others). Also, we considered respondents’ wealth index based on household’s ownership of selected assets, such as television, materials used for housing construction and types of water and sanitation facilities (poorest, poorer, middle, richer and richest). We coded poorest and poorer as “poor” and richer and richest as “rich.” Current marital status was coded as “married” and “not married.” Not married constitutes never married, divorced, widowed and separated. Ethnicity was coded as Mende, Temne, Limba and other tribes.

### Data analysis

We used SPSS version 28, using the complex sample command to conduct descriptive and inferential analyses to account for weighting and complex sampling design. We presented categorical variables using unweighted frequencies and weighted percentages. Bivariate analysis (Pearson chi-square) was conducted to identify characteristics significantly associated with our outcome variable (homebirth). We created two regression models. We included only the main independent variable (women’s empowerment parameters) in the first model. Outcome of this model (model 1) was presented as crude odds ratio (cOR). Explanatory variables in the bivariate analysis with p-value ≤ 0.25 were entered into the multivariable binary logistic regression model (model 2), presented as adjusted odd ratio (aOR) with corresponding 95% confidence intervals (CI). Statistical significance was considered if *p* < 0.05. Using the variance inflation factor (VIF), we tested for multicollinearity among independent variables. We found no evidence of multicollinearity among the independent variables (Min VIF = 1.018, Max VIF = 2.536) (Additional file 1).

## Results

### Sociodemographic characteristics

provides the sociodemographic characteristics of women who gave birth and the proportion. Approximately two-thirds of women, who gave birth to their last child at home in the five years preceding the survey were aged 20–34 years (n = 4814; 65.9%), lived in rural areas (n = 4904; 61.9%) (Table 1). Three-fourth were married (n = 5748; 76.9%) while one in four had eight or more ANC visits (n = 1677; 25.0%). Approximately one in every two women considered the distance to a health facility a big problem (n = 3668; 47.1%) and were against the idea of IPV against women (n = 7708; 50.1%). Knowledge level was low in approximately one-third (n = 5051; 29.0%) and approximately four in nine women had low decision-making power (n = 4087; 42.7%) (Table 1). Three in every 20 women gave birth at home (n = 1177; 15.3%).

**Table 1:** Socio-demographic and obstetric characteristics of respondents (n = 7377), Sierra Leone 2019 Demographic Health Survey (SLDHS2019)

Characteristics	Variable	Total n^a^(%)^b^
Home Birth	Yes	1177(15.3)
	No	6200(84.7)
Age	15–19	601(8.2)
	20–34	4814(65.9)
	35–49	1962(25.9)
Region	Eastern	1475(21.0)
	Northern	1713(19.6)
	North-western	1379(18.8)
	Southern	1794(20.4)
	Western	1016(20.2)
Residence	Urban	2473(38.1)
	Rural	4904(61.9)
Religion	Christianity/None	1531(21.3)
	Islam	5846(78.7)
Wealth Index	Poor	3421(42.8)
	Middle	1563(20.3)
	Rich	2393(36.9)
Current Marital Status	Married	5748(76.9)
	Not Married^c^	1629(23.1)
Ethnicity	Limba	619(8.8)
	Mende	2484(34.5)
	Temne	2334(36.6)
	Others	1509(20.1)
Husband/partner’s education level	No education	3479(54.0)
	Primary	455(7.7)
	Secondary	1550(27.4)
	Higher	441(7.8)
	Don’t know	165(3.1)
Husband/partner’s occupation	Not working/ Don’t know	312(5.7)
	Working/Had a job	5778(94.3)
Sex of Household head	Male	5629(75.3)
	Female	1748(24.7)
Parity	1–5	6145(83.9)
	> 5	1232(16.1)
Ever had a terminated pregnancy	Yes	546(7.6)
	No	6831(92.4)
Wanted last child	Wanted then	6054(80.9)
	Wanted later	1056(15.4)
	Wanted no more	267(3.7)
Number Antenatal care visit	No Antenatal visit	101(1.7)
	1–3 visits	573(8.8)
	4–7 visits	4189(64.5)
	8 or more visits	1677(25.0)
Getting medical help for self: getting permission to go to hospital	Big problem	1998(24.9)
	Not a big problem	5379(75.1)
Getting medical help for self: getting money needed for treatment	Big problem	5329(70.1)
	Not a big problem	2048(29.9)
Getting medical help for self: distance to health facility	Big problem	3668(47.1)
	Not a big problem	3709(52.9)
Covered by health insurance	Yes	247(3.8)
	No	7130(96.2)
Women’s knowledge level	Low	5051(29.0)
	Medium	6356(41.6)
	High	4159(29.4)
Decision making power	Low	4087(42.7)
	Medium	2315(22.4)
	High	3435(34.9)
**Women’s level of tolerance to the idea of intimate partner violence (wife beating)**	No/low	7708(50.1)
	Medium	6611(41.6)
	High	1255(8.3)
Currently Working	Yes	5749(77.0)
	No	1628(23.0)

Note a = Un weighted count

b = weighted percentage

C = single, divorced and separated

### Bivariate association between sociodemographic and obstetric characteristics and place of birth

Socio-demographic and obstetric characteristics that showed significant associations with place of birth included age (*p* < 0.001), region (*p* < 0.001), residence (*p* < 0.001) wealth index (*p* < 0.001), marital status (*p* = 0.002), number antenatal care visits (*p* < 0.001) and parity (*p* < 0.001) (Table 2).

**Table 2:** Bivariate analysis Socio-demographic and obstetric characteristics of respondents (n = 7377) and homebirth using Sierra Leone 2019 Demographic Health Survey (SLDHS2019)

Characteristics	Variable	Home birth		Chi-square, p-values
		Yes n^a^(%)^b^	No n^a^(%)^b^	
Age	15–19	79(6.7)	522(8.4)	31.55, *p* < 0.001
	20–34	709(60.8)	4105(66.9)	
	35–49	389(32.5)	1573(24.7)	
Region	Eastern	108(10.0)	1367(23.0)	350.22, *p* < 0.001
	Northern	310(19.0)	1403(19.7)	
	North-western	404(37.8)	975(15.4)	
	Southern	229(17.4)	1565(20.9)	
	Western	126(15.8)	890(21.0)	
Residence	Urban	248(26.4)	2225(40.3)	77.62, *p* < 0.001
	Rural	929(73.6)	3975(59.7)	
Religion	Christianity/None	184(15.4)	1347(22.4)	27.27, *p* < 0.001
	Islam	993(84.6)	4853(77.6)	
Wealth Index	Poor	692(53.5)	2729(40.9)	79.84, *p* < 0.001
	Middle	241(20.7)	1322(20.2)	
	Rich	244(25.8)	2149(38.9)	
Current Marital Status	Married	985(81.8)	4763(76.0)	18.01, *p* = 0.002
	Not Married^c^	192(18.2)	1437(24.0)	
Ethnicity	Limba	116(9.4)	503(8.7)	226.25, *p* < 0.001
	Mende	156(14.7)	2328(38.0)	
	Temne	515(51.7)	1819(33.9)	
	Others	282(24.2)	1227(19.4)	
Husband/partner’s education level	No education	713(66.0)	2766(51.7)	72.72, *p* < 0.001
	Primary	61(6.3)	394(8.0)	
	Secondary	189(21.5)	1361(28.6)	
	Higher	35(4.1)	406(8.5)	
	Don’t know	20(2.2)	145(3.3)	
Husband/partner’s occupation	Not working/ Don’t know	39(4.0)	273(6.0)	6.02, *p* = 0.028
	Working/Had a job	979(96.0)	4799 (94.0)	
Sex of Household head	Male	912(74.9)	4717(75.4)	0.14, *p* = 0 0.788
	Female	265(25.1)	1483(24.6)	
Parity	1–5	927(80.0)	5218(84.6)	15.03, *p* < 0.001
	> 5	250(20.0)	982(15.4)	
Ever had a terminated pregnancy	Yes	99(8.8)	447(7.3)	3.04, *p* = 0 0.172
	No	1078(91.2)	5753(92.7)	
Wanted last child	Wanted then	978(81.5)	5076(80.8)	3.97, *p* = 0 0.283
	Wanted later	149(14.0)	907(15.6)	
	Wanted no more	50(4.5)	217(3.6)	
Number Antenatal care visit	No Antenatal visit	41(3.2)	60(1.5)	97.91, *p* < 0.001
	1–3 visits	194(15.9)	379(7.4)	
	4–7 visits	599(56.5)	3590(66.0)	
	8 or more visits	238(24.3)	1439(25.1)	
Getting medical help for self: getting permission to go to hospital	Big problem	339(27.2)	1659(24.5)	3.52, *p* = 0 0.240
	Not a big problem	838(72.8)	4541(75.5)	
Getting medical help for self: getting money needed for treatment	Big problem	940(78.1)	4389(68.7)	40.81, *p* < 0.001
	Not a big problem	237(21.9)	1811(31.3)	
Getting medical help for self: distance to health facility	Big problem	776(62.2)	2892(44.4)	120.11, *p* < 0.001
	Not a big problem	401(37.8)	3308(55.6)	
Covered by health insurance	Yes	50(4.0)	197(3.7)	0 0.18, *p* = 0 0.785
	No	1127(96.0)	6003(96.3)	
Women’s knowledge level	Low	586(46.2)	2161(31.8)	116.70, *p* < 0.001
	Medium	465(40.8)	2639(43.5)	
	High	126(13.0)	1397(24.7)	
Decision making power	Low	452(44.1)	2174(44.2)	10.70, *p* = 0.058
	Medium	261(26.1)	1173(21.8)	
	High	305(29.8)	1725(34.0)	
**Women’s level of tolerance to the idea of intimate partner violence (wife beating)**	No/low	428(36.1)	2994(48.5)	58.93, *p* < 0.001
	Medium	638(54.4)	2717(43.3)	
	High	111(9.4)	489(8.2)	
Currently Working	Yes	979(82.0)	4770(76.1)	18.29, *p* = 0.002
	No	198(18.0)	1430(23.9)	

Note a = Un weighted count

b = weighted percentage

c = single, divorced and separated

Just over half of the women who gave birth at home were poor (692; 53.5%), while one-fourth were in the rich quantile (n = 244; 25.8%). In addition, nearly three-fourth who decided to give birth at home lived in rural areas (n = 929; 73.6%). Approximately three in eight women who gave birth at home were against the idea of IPV against women (n = 428; 36.1%), while close to half had low knowledge level (n = 586; 46.2%), and low decision-making power (n = 452; 44.1%) and approximately eight in ten were working (n = 979; 82.0%).

### Association of women’s empowerment, other sociodemographic and obstetric determinants and choice of homebirth

Women with low [aOR2.04; 95% CI 1.43–2.92] and medium levels of knowledge [aOR 1.44; 95% CI 1.05–1.97] had higher odds of giving birth at home compared to those with high levels of knowledge (Table 3). Also, women who had no power to refuse the idea of IPV against women were more likely to have had homebirth compared to those against the idea [aOR 1.38; 95% CI 1.09–1.74]. However, no significant association was observed between women’s decision-making power and home birth [aOR 1.11; 95% CI 0.86–1.41]. In addition, women with no [aOR 2.71; 95% CI 1.34–5.46) and less than four antenatal care visits [aOR 2.08; 95% CI1.51-2.88] and who considered the distance to a health facility as a major problem [aOR 1.95; 95% CI 1.49–2.56] were more likely to have had a home birth. On the other hand, we observed that women from the Mende ethnic group were less likely than those from other ethnic minority groups to give birth at home [aOR 0.26; 95% CI 0.16–0.43].

**Table 3:** Determinants of Home birth in Sierra Leone, Sierra Leone 2019 Demographic Health Survey (SLDHS2019)

		Model 1	Model 2
Characteristics	Variable	COR (95%CI)	AOR (95%CI)
Women’s knowledge level	High	1	**1**
	Low	2.76(2.10–3.62)	**2.04(1.43–2.92)**
	Medium	1.78(1.38–2.29)	**1.44(1.05–1.97)**
Decision making power	High	1	**1**
	Low	1.138(0.92–1.41)	1.11(0.86–1.41)
	Medium	1.36(1.09–1.70)	1.16(0.88–1.53)
**Women’s level of tolerance to the idea of intimate partner violence (wife beating)**	No/low	1	**1**
	Medium	1.69(1.39–2.05)	**1.38(1.09–1.74)**
	High	1.54(1.13–2.10)	1.14(0.80–1.62)
Labour force Participation	Yes	1	1
	No	0.70(0.56–0.88)	1.00(0.76–1.33)
Age	15–19		1
	20–34		1.04(0.70–1.55)
	35–49		1.20(0.77–1.89)
Region	Western		1
	Eastern		**0.39(0.21–0.71**)
	Northern		0.65(0.38–1.12)
	North-western		1.45(0.85–2.44)
	Southern		1.30(0.74–2.28)
Residence	Urban		1
	Rural		1.19(0.80–1.75)
Religion	Christianity/None		1
	Islam		0,85(0.61–1.17)
Wealth Index	Rich		1
	Poor		1.25(0.83–1.88)
	Middle		1.19(0.79–1.80)
Marital status	Not Married		1
	Married		1.16(0.62–2.01)
Ethnicity	Others		1
	Limba		0.73(0.45–1.19)
	Mende		**0.26(0.16–0.43)**
	Temne		0.90(0.66–1.22)
Husband/partner’s education level	Higher		1
	No formal education		1.40(0.83–2.36)
	Primary school		1.01(0.56–1.85)
	Secondary school		1.18(0.70-2.00)
	Don’t Know		1.18(0.54–2.58)
Husband/partner’s occupation	Working/Had a job		1
	Did not work/Don’t Know		0.83(0.53–1.29)
Parity	1–5		1
	> 5		0.88(0.69–1.12)
Ever had a terminated pregnancy	Yes		1
	No		0.84(0.62–1.13)
Number of Antenatal visits	8 or more visits		1
	No Antenatal visit		**2.71(1.34–5.46)**
	1–3 visits		**2.08(1.51–2.88)**
	4–7 visits		0.88(0.69–1.12)
Getting medical help for self: getting permission to go to hospital	Not a big problem		1
	Big Problem		**0.72(0.55–0.94)**
Getting medical help for self: getting money needed for treatment	Not a big problem		1
	Big Problem		1.02(0.77–1.35)
Getting medical help for self: distance to health facility	Not a big problem		1
	Big Problem		**1.95(1.49–2.56)**

## Discussion

Sierra Leone is considered one of the countries with the highest MMRs and several individual, community and health system factors are responsible. One factor may be women’s autonomy and self-determination relating to their health. Homebirth without a skilled birth attendant has been associated with perinatal and neonatal mortality [3, 36]. Three in every 20 (15%) Sierra Leonean women chose to give birth of the last child at home, a reduction from 44% to 2013 and 72% in 2008 [37]. Our finding is consistent with reports from Benin [16] but lower than the reported prevalences in Mali, Tanzania, Senegal and Ethiopia [18, 20, 26, 38]. This may be explained by increased investments by the government and its partners to improve access to maternal and child health care at policy and care provision levels to reduce the seemingly high MMR [8, 39]. The establishment of the free healthcare policy in 2010, for pregnant and lactating mothers as well as for under-fives, the health Sector Recovery Plan and the Basic Package of Essential Health Care have led to an increase in the number of maternity care facilities and training of more midwives and maternal and child healthcare providers [39, 40]. Such gains may have contributed to the reduction of home births. Yet, MMR is still high in Sierra Leone (1). Such a discrepancy between maternity care utilisation and outcome has been attributed to the quality of care in health facilities with misdiagnosis and nonadherence to standard treatment guidelines considered as contributing factors (10).

On the other hand, our finding suggests that much still needs to be done, despite the success in reducing the proportion of homebirth over the years. The 2013–2016 Ebola outbreak is known to have further weakened the Sierra Leone healthcare system, which would have prevented the further reduction of homebirth and the overall high MMR and infant mortality rate [41, 42]. In addition to the impact of Ebola, our study showcases several sociodemographic and obstetric characteristics of Sierra Leonean women that are potential risk factors for their choice of home over institutional birth.

In line with similar studies in other African countries [16, 19, 20, 26, 38], a recent cross-sectional study in Kailahun District, Sierra Leone [43], reported that women with low levels of knowledge were more likely to give birth at home compared to those with higher knowledge levels. The fact that being illiterate and having no or less media access is linked to reduced exposure to and understanding health information, especially the importance of preventing complications during childbirth and postpartum, may explain our findings. These reflect lack of access to information to make informed decisions about pregnancy and labour, including danger signs and complications. For instance, having poor knowledge of obstetric complications is a determinant of homebirth [17, 44] and having at least high school education has been shown to promote self-efficacy and awareness of health care and proper appraisal of health information to make informed decisions [45, 46]. A qualitative study in rural Sierra Leone revealed that women considered home birth normal and only sought care in a hospital if a problem occurred [15]. Similar findings have been reported in Ghana [33], Benin [47] and Ethiopia [17]. This underscores mass media’s importance in promoting childbirth in health facilities.

Consistent with similar studies [5, 26, 48], women who seem to have no power to resist the idea of IPV had higher odds of giving birth at home. This may be explained by women not having the space or support at legislative and community levels that empowers them to stand up against IPV. In most African societies, legislative structures and culture do not provide an opportunity for girls to go to school or own property to be intellectually and economically empowered to stand against IPV. In Sierra Leone women are less likely to go to school and less exposed to economic opportunities than men [12]. IPV may be considered a social norm passed down from one generation to the other [49, 50]. This is prevalent in rural settings where the patriarchal system still exists. Patriarchy in Africa puts women in marginalised and subordinate positions in society [51]. Women’s activities are restricted to procreation and household chores relying solely on their male counterpart to make decisions on education, finances, and health care [51].

Studies have shown that women who witnessed IPV during childhood are likely to experience it when they are mature and lack power to resist gender-based violence as the patriarchal system demands women to be submissive and condones IPV [49]. Our study did not explore why women seem to condone IPV, and its association with homebirth warrants further study.

Consistent with literature from other African countries [16, 17, 38, 52], women with less than four ANC visits were more likely to give birth at home than those with eight or more visits. Given that ANC serves as a point of contact for pregnant women with the healthcare system, it is an opportunity to learn about the associated risks of childbirth at home and the need for facility-based childbirth. In line with WHO, the current National Reproductive, Maternal, Newborn, Child and Adolescent Health Policy of Sierra Leone recommends that pregnant women have eight visits, starting within the first 12 weeks of gestation [7]. Women who considered distance to health facilities a big problem were more likely to give birth at home. This is supported by previous African studies [17, 21, 38] and lends credence to the fact that women who are in labour are unable to walk to a health facility that is far away, especially in rural areas where poor road infrastructure is common [13]. Distance to health facilities, including lack of transport and perceived negative behaviour of hospital staff played a crucial role in women’s decision to give birth at home [14].

### Strengths and limitations

Our findings are representative of women of reproductive age in Sierra Leone since DHS uses a representative sample. We cannot infer, however, any causality between outcome and independent variables, given the cross-sectional nature of our study. A tendency for recall bias may exist since data were collected among women who had given birth within five years preceding the survey.

### Study implications

Our findings underscore the need for the Sierra Leone Ministry of Health and Sanitation and other stakeholders to continue implementing strategies to strengthen the coverage of ANC visits and promote birth preparedness and complication readiness using a multidisciplinary approach. Women’s empowerment through education to promote health-seeking behaviour and decision-making capacity should be an area of focused attention. Maternal health interventions, such as Free Healthcare Initiative (FHCI), primarily focus on improving access to maternity care by removing user fees, building more health facilities and training more health personnel. Although these strategies are essential, studies have identified that care may not be accessed by women who lack power in unequal gender relations and are subject to societal norms that favour IPV and low female participation in decision-making regarding health [53]. Thus, to improve access, it is equally vital for maternal health interventions to focus on promoting education among women and girls, improving access to sexual and reproductive health information and removing societal norms and unequal power relations [54]. Community-based interventions that use a peer-led approach in which peer educators are used as conduits to transfer knowledge among women and to help provide a supportive environment at household and community levels. That can be effective to empower women to demand access to maternal and child health care [55].

## Conclusion

Homebirth in Sierra Leone is still a maternal health issue despite the government’s and its partners’ success in reducing its prevalence over the years. Women with low knowledge levels, who lack power to resist IPV, had less than four ANC visits and considered distance to health facilities a significant problem, had higher odds of giving birth at home. Our findings suggest that improving knowledge and preventing gender-based violence are women’s empowerment parameters that need policy and practice considerations. Importantly, Government and partners in Sierra Leone need to implement maternal health interventions that focus on improving knowledge through education and removing societal norms and unequal power relations that hinder women’s empowerment.

### Electronic supplementary material

Below is the link to the electronic supplementary material.

Supplementary Material 1

## Data Availability

The dataset informing the findings of this study is publicly available at https://dhsprogram.com/data/available-datasets.cfm.

## Abbreviations

SLDHS: Sierra Leone Demographic and Health Survey
WHO: World Health Organization
sSA: Sub-Saharan Africa
ANC: Antenatal Care
FHCI: Free Healthcare Initiative
EA: Enumeration Areas
aOR: Adjusted Odd Ratio
IPV: Intimate Partner Violence
