# Supplementary material for: Do disempowered childbearing women give birth at home in Sierra Leone? A secondary analysis of the 2019 Sierra Leone demographic health survey
Source: BMC Pregnancy Childbirth. 2023 Nov 22;23:810. doi: 10.1186/s12884-023-06126-y (PMC10664373; doi:10.1186/s12884-023-06126-y)
Supplement: Supplementary file 1 — Supplementary Material 1 [file 12884_2023_6126_MOESM1_ESM.docx]

**Additional file 1: Collinearity Statistics**

| **Collinearity Statistics** | | |
| --- | --- | --- |
|  | Tolerance | VIF |
| Region | 0.925 | 1.082 |
| Type of place of residence | 0.418 | 2.390 |
| Religion | 0.930 | 1.075 |
| Ever had a terminated pregnancy | 0.982 | 1.018 |
| Husband/partner's education level | 0.838 | 1.194 |
| Respondent currently working | 0.892 | 1.121 |
| Husband/partner's occupation (grouped) | 0.952 | 1.050 |
| Parity | 0.761 | 1.314 |
| Number of Antenatal visits | 0.956 | 1.046 |
| Age Categorised | 0.742 | 1.347 |
| Wealth Index | 0.394 | 2.536 |
| Ethnicity | 0.923 | 1.084 |
| Marital Status | 0.952 | 1.050 |
| Wife Beating | 0.936 | 1.068 |
| Knowledge level | 0.726 | 1.377 |
| Decision making power | .912 | 1.096 |
| Getting medical help for self: getting permission to go hospital | 0.819 | 1.221 |
| Getting medical help for self: getting money needed for treatment | 0.763 | 1.311 |
| Getting medical help for self: distance to health facility | 0.691 | 1.447 |
